# Oestrogen receptor-**α** variant mRNA expression in primary human breast tumours and matched lymph node metastases

**DOI:** 10.1038/sj.bjc.6690156

**Published:** 1999-02

**Authors:** E Leygue, R E Hall, H Dotzlaw, P H Watson, L C Murphy

**Affiliations:** Department of Biochemistry and Molecular Biology, University of Manitoba, Winnipeg, MB, Canada; Department of Surgery, Flinders University of South Australia, Bedford Park, SA, Australia; Department of Pathology, University of Manitoba, Winnipeg, MB, Canada

**Keywords:** oestrogen receptor-α, variants, breast cancer, metastasis

## Abstract

We have shown previously that the relative expression of a truncated oestrogen receptor-α variant mRNA (ER clone 4) is significantly increased in axillary node-positive primary breast tumours compared with node-negative tumours. In this study, we have examined the relative expression of clone 4-truncated, exon 5-deleted and exon 7-deleted oestrogen receptor-α variant mRNAs in 15 primary breast tumour samples and in synchronous axillary lymph node metastases. Overall, there were no significant differences between the primary tumours and the matched metastases in the relative expression of these three specific variant mRNAs. Furthermore, the pattern of all deleted oestrogen receptor-α variant mRNAs appeared conserved between any primary and its matched secondary tumour. *© 1999 Cancer Research Campaign*


*British Journal of Cancer* (1999) **79**(5/6), 978–983
© 1999 Cancer Research Campaign
Article no. bjoc.1998.0156

### Oestrogen receptor-α variant mRNA expression in primary human breast tumours and matched lymph node metastases

**E Leygue[1], RE Hall[2], H Dotzlaw[1], PH Watson[3] and LC Murphy[1]**

[1]Department of Biochemistry and Molecular Biology, University of Manitoba, Winnipeg, MB, Canada; [2]Department of Surgery, Flinders University of South Australia, Bedford Park, SA, Australia; [3]Department of Pathology, University of Manitoba, Winnipeg, MB, Canada

**Summary** We have shown previously that the relative expression of a truncated oestrogen receptor-α variant mRNA (ER clone 4) is significantly increased in axillary node-positive primary breast tumours compared with node-negative tumours. In this study, we have examined the relative expression of clone 4-truncated, exon 5-deleted and exon 7-deleted oestrogen receptor-α variant mRNAs in 15 primary breast tumour samples and in synchronous axillary lymph node metastases. Overall, there were no significant differences between the primary tumours and the matched metastases in the relative expression of these three specific variant mRNAs. Furthermore, the pattern of all deleted oestrogen receptor-α variant mRNAs appeared conserved between any primary and its matched secondary tumour.

**Keywords:** oestrogen receptor-α variants; breast cancer; metastasis

Multiple oestrogen receptor-α (ER) mRNA species have been identified in human breast cancer samples (Dowsett et al, 1997; Murphy et al, 1997*a*, *b*). The significance of these variant transcripts remains unclear. Although the ability to detect variant ER proteins encoded by such variant transcripts remains controversial (Park et al, 1996; Desai et al, 1997; Huang et al, 1997), alteration of expression of some variant ER mRNAs has been found to occur during both breast tumorigenesis (Leygue et al, 1996*a*, *b*) and breast cancer progression. With regard to the latter, we showed previously that the expression of the truncated, clone 4 variant (C4) ER mRNA (Dotzlaw et al, 1992) was significantly increased relative to wild-type (WT) ER mRNA in a group of primary breast tumours with multiple poor prognostic features compared with a group of primary breast tumours with good prognostic features (Murphy et al, 1995). The ‘poor’ prognostic features were defined as the presence of lymph node metastases at the time of surgery, large tumour size, lack of progesterone receptor (PR) expression and high S-phase fraction, while ‘good’ prognostic features were lack of nodal involvement, small tumour size, PR positivity and low S-phase fraction. In the same study, the relative expression of clone 4 ER variant mRNA was significantly higher in primary breast tumours that were PR– than in those that were PR+ (Murphy et al, 1995). This suggested that altered ER variant expression may be a marker of a more aggressive phenotype and lack of endocrine sensitivity in human breast cancer. As a prerequisite to addressing such a possibility, we have investigated the pattern of ER variant expression in a cohort of primary tumours and their matched, concurrent lymph node metastases.

*Received 20 April 1998*
*Revised 3 July 1998*
*Accepted 14 July 1998*

*Correspondence to:* LC Murphy

### MATERIALS AND METHODS

### Tumour selection and RNA isolation

Sections from 15 frozen primary human breast tumour samples and their matched frozen lymph node metastases were provided by the Manitoba Breast Tumour Bank (Winnipeg, MB, Canada). For the primary tumour samples, the ER levels, determined by ligand-binding assays, ranged from 0.8 fmol $mg^{-1}$ protein to 89 fmol $mg^{-1}$ protein with a median value of 17.5 fmol $mg^{-1}$ protein. Thirteen tumours were ER+ and two were ER– (ER+ was defined as >3 fmol $mg^{-1}$ protein). PR levels determined by ligand-binding assays ranged from 2.9 fmol $mg^{-1}$ protein to 112 fmol $mg^{-1}$ protein with a median value of 12.6 fmol $mg^{-1}$ protein. Nine tumours were PR+ and 6 were PR– (PR+ was defined as > 10 fmol $mg^{-1}$ protein). ER and PR values were available for only four of the lymph node metastases and the ER and PR status as defined by ligand binding did not differ from their matched primary tumour. RNA was extracted from the sections using Trizol reagent (Gibco/BRL, Ontario, Canada) according to the manufacturer’s instructions.

For validation of triple-primer polymerase chain reactions (TP-PCR) by comparison with RNAase protection assays, a second cohort of human breast tumour specimens (25 cases) was also obtained from the Manitoba Breast Tumour Bank. Twenty of these tumours were ER+, as determined by ligand-binding assay, with values ranging from 4.5 to 311 fmol $mg^{-1}$ protein (median 93 fmol $mg^{-1}$). The five remaining cases were ER–, with values ranging from 0 to 1.8 fmol $mg^{-1}$ protein (median 0.9 fmol $mg^{-1}$). Total RNA was extracted from frozen tissues using guanidinium thiocyanate as previously described (Murphy and Dotzlaw, 1989). The integrity of the RNA was confirmed by denaturing gel electrophoresis as previously described (Murphy and Dotzlaw, 1989).

### RNAase protection assay

Antisense riboprobes spanning the point at which the C4 ER mRNA sequence diverges from the WT ER mRNA sequence (Dotzlaw et al, 1992) were synthesized as previously described (Dotzlaw et al, 1990). The level of C4 ER mRNA and WT ER mRNA in 10 μg of total RNA was determined using an RNAase Protection Assay kit (RPA II, Ambion, Austin, TX, USA) following the manufacturer's instructions. Briefly, RNA was denatured at 80°C for 5 min in the presence of $5 \times 10^5$ d.p.m. $^{32}$P-labelled riboprobe, then hybridized at 42°C for 16 h. Following RNAase digestion, samples were electrophoresed on 6% acrylamide gels containing 7 M urea, dried and autoradiographed.

To quantify C4 and WT ER mRNAs within breast tumour samples, a standard curve was established in each assay. C4 and WT ER mRNAs (30, 125, 500 pg C4 RNA and 125, 500, 2000 pg WT ER RNA) synthesized using T7 RNA polymerase were purified on a Sephadex G-50 column and quantitated spectrophotometrically. WT ER RNA was transcribed from linearized pHEO, which contains the entire WT ER coding sequence but is missing the 3′-untranslated portion of the ER mRNA [(kindly provided by P Chambon, Strasbourg, France (Green et al, 1986)]. Full-length C4 RNA was transcribed from linearized pSK-C4 (Dotzlaw et al, 1992). Standard RNAs were analysed together in the same assay as the breast tumour mRNAs. Bands corresponding to the C4 ER mRNA and WT ER mRNA protected fragments were excised from the gel and counted after addition of 5 ml scintillant (ICN Pharmaceuticals, Inc., Irvine, CA, USA) in a scintillation counter (Beckman Instruments, Inc., Fullerton, CA, USA). For each sample, absolute amounts of C4 and WT ER mRNA were determined from the standard curve.

### Reverse transcription, PCR and triple-primer (TP) PCR

For each sample, 1 μg of total RNA was reverse transcribed in a final volume of 15 μl as described previously (Leygue et al, 1996*a*). One microlitre of the reaction mixture was taken for subsequent amplification.

The primers and PCR conditions for the long-range PCR were as previously described (Leygue et al, 1996*c*). The primers and PCR conditions for measuring the relative expression of exon 5-deleted and exon 7-deleted ER transcripts relative to WT ER transcripts were as previously described (Leygue et al, 1996*a*).

The TP-PCR conditions were similar to those previously described (Leygue et al, 1996*b*) with minor modifications. ERU (5′-TGTGCAATGACTATGCTTCA-3′, sense, located in WT ER exon 2; 792–811, as numbered in Green et al, 1986) and ERL (5′-GCTCTTCCTCCTGTTTTTAT-3′, antisense, located in WT ER exon 3; 940–921) primers allowed amplification of a 149-bp fragment corresponding to WT ER mRNA. The C4-specific primer (C4L, 5′-TTTCAGTCTTCAGATACCCCAG-3′, antisense; 1336–1315, as numbered in Dotzlaw et al, 1992) spans the only region of the C4 unique sequence that does not have any homology with repetitive LINE-1 sequences (Dotzlaw et al, 1992). ERU and C4L allowed amplification of a 536-bp fragment corresponding specifically to C4 ER variant mRNA.

PCR amplifications were performed in a final volume of 10 μl in the presence of 20 mM Tris-HCl (pH 8.4), 50 mM potassium chloride,

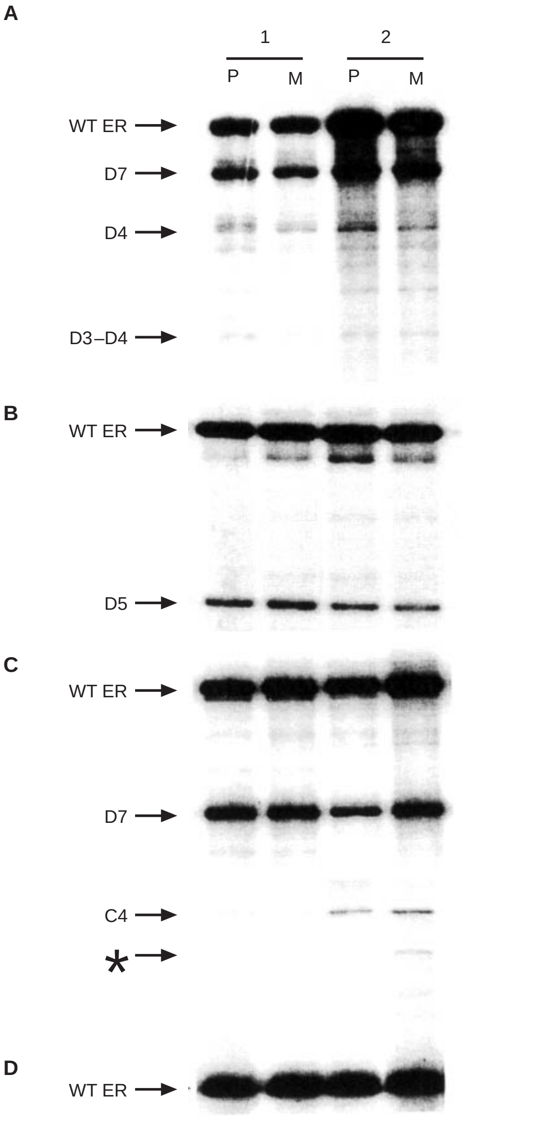

**Figure 1** (**A**) Autoradiogram of long-range RT-PCR (Leygue et al, 1996*c*) results from two samples of primary breast tumours (P) and their matched concurrent lymph node metastase (M). WT ER is the expected product corresponding to the WT ER mRNA; D7 is the expected product corresponding to the exon 7-deleted ER variant mRNA; D4 is the expected product for the exon 4-deleted ER mRNA; D3-4 is the expected product for the exon 3+4-deleted ER mRNA; D4/7 is the expected product for the exon 4+7-deleted ER mRNA. (**B**) Autoradiogram of RT-PCR results from two samples of primary breast tumours (P) and their matched concurrent lymph node metastase (M). D5 is the expected product corresponding to the exon 5-deleted ER variant mRNA. WT ER is the expected product corresponding to the WT ER mRNA. (**C**) Autoradiogram of RT-PCR results from two samples of primary breast tumours (P) and their matched concurrent lymph node metastase (M). D7 is the expected product corresponding to the exon 7-deleted ER variant mRNA. WT ER is the expected product corresponding to the WT ER mRNA. (**D**) Autoradiogram of TP-PCR results from two samples of primary breast tumours (P) and their matched concurrent lymph node metastase (M). C4 is the expected product corresponding to the clone 4 ER variant mRNA. WT ER is the expected product corresponding to the WT ER mRNA. *Band coamplified with C4 and WT ER and shown to correspond to an exon 2-duplicated ER variant mRNA

2 mM magnesium chloride, 0.2 mM dATP, 0.2 mM dTTP, 0.2 mM dGTP, 0.2 mM dCTP, 4 ng $\mu l^{-1}$ of each primer (ERU, ERL and C4L), 0.2 units of *Taq* DNA polymerase (Gibco-BRL) and 1 μCi of [α-$^{32}$P] dCTP (3000 Ci $mmol^{-1}$, ICN Pharmaceuticals, Irvine, CA, USA). Each PCR consisted of 30 cycles (1 min at 94°C, 30 s at 60°C and

*© Cancer Research Campaign 1999*

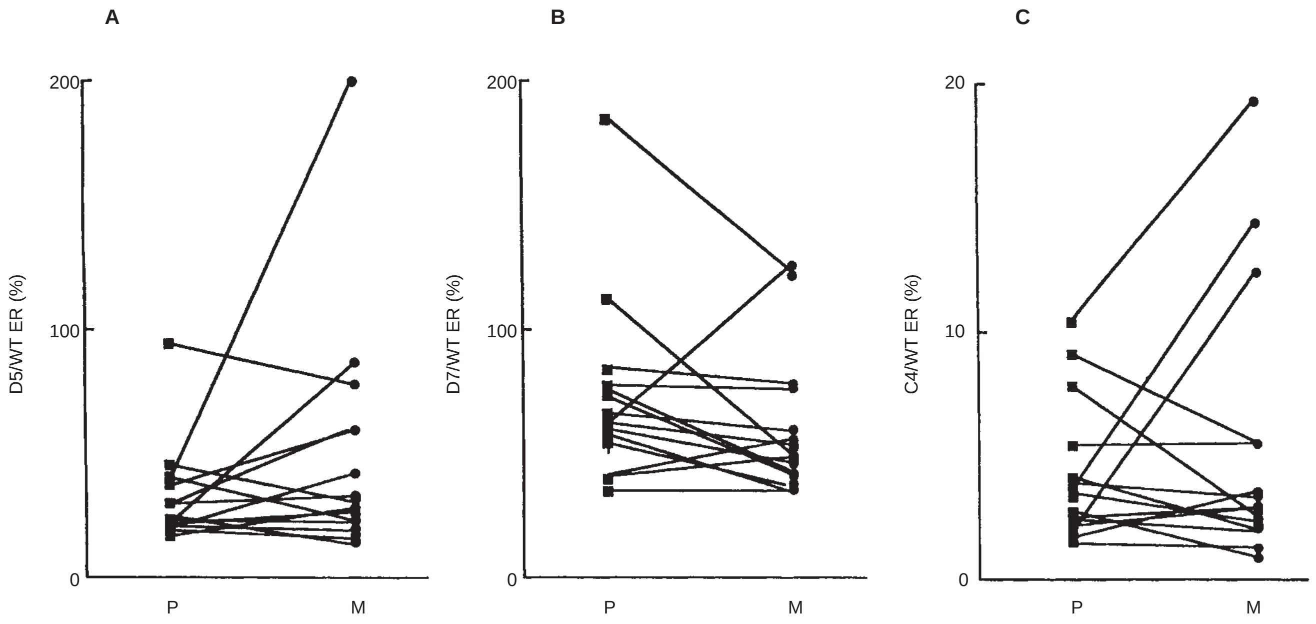

**Figure 2** (**A**) Quantitative comparison of the relative expression of exon 5-deleted variant ER mRNA in primary (P) human breast tumours and their concurrent matched lymph node metastases (M). For each sample, the mean of three independent measures of exon 5-deleted ER relative expression expressed as a percentage of the corresponding WT ER signal was determined as described in the Materials and methods section. (**B**) Quantitative comparison of the relative expression of exon 7-deleted variant ER mRNA in primary (P) human breast tumours and their concurrent matched lymph node metastases (M). For each sample, the mean of three independent measures of exon 7-deleted ER relative expression expressed as a percentage of the corresponding WT ER signal was determined as described in the Materials and Methods section. (**C**) Quantitative comparison of the relative expression of clone 4 variant ER mRNA in primary (P) human breast tumours and their concurrent matched lymph node metastases (M). For each sample, the mean of three independent measures of clone 4 relative expression expressed as a percentage of the corresponding WT ER signal was determined as described in the Materials and Methods section

1 min at 72°C) using a thermocycler (Perkin Elmer). Four microlitres of the reaction mix was then denatured by addition of 6 µl of 80% formamide buffer and boiling before electrophoresis on 6% polyacrylamide gels containing 7 M urea (PAGE). Following electrophoresis, the gels were dried and exposed to Kodak XAR Film at –70°C with two intensifying screens for 2 h.

### Quantification of RT-PCR and TP-PCR

Bands corresponding to the variant ER mRNA and WT ER mRNA were excised from the gel and counted after addition of 5 ml of scintillant in a scintillation counter. The variant signal was expressed as a percentage of the WT ER signal. It should be noted that the percentage obtained reflects the relative ratio of the variant to WT ER RT-PCR product and does not provide absolute initial mRNA levels. Validation of this approach was described previously (Daffada et al, 1994, 1995; Leygue et al, 1996*a*, *b*). At least two independent PCR assays were performed for each sample in the comparison of RNAase protection assay with TP-PCR assays. For assessment of matched primary and secondary breast tumour samples, at least two and in most cases three independent PCR reactions were performed and the mean determined.

The statistical significance of differences in the relative levels of expression of any single ER mRNA variant between primary tumour and lymph node metastasis was determined using the Wilcoxon signed-rank test.

### RESULTS

### Determination of the pattern of exon-deleted ER variant mRNA expression

Multiple ER variant mRNAs have been shown to be expressed in any one breast tissue sample (Leygue et al, 1996*a*; Murphy et al, 1997*a*, *b*). To investigate the pattern of multiple exon-deleted ER variant expression between primary breast tumours and their matched lymph node metastases, a long-range RT-PCR approach was used. This approach, based on the competitive amplification of wild-type and exon-deleted ER variant cDNAs, using primers annealing within exons 1 and 8, allows the evaluation of the relative pattern of expression of all exon-deleted ER variant transcripts present in any individual sample (Leygue et al, 1996*c*; Fasco, 1997). Typical results are shown in Figure 1A. The pattern of deleted ER mRNA expression between any one primary tumour and its matched lymph node metastasis was conserved.

### Determination of the relative expression of exon 5-deleted and exon 7-deleted ER variant mRNA expression

Using a previously validated semiquantitative PCR approach (Leygue et al, 1996*a*), the measurement of the relative expression of specific individual exon-deleted ER variant mRNAs was also undertaken. Specifically, the relative expressions of exon 5-deleted

 © Cancer Research Campaign 1999

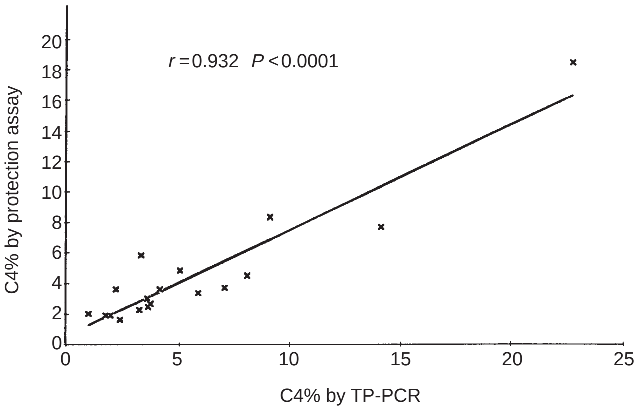

**Figure 3** Linear regression analysis of clone 4 expression (expressed as a percentage of the corresponding WT ER expression) as determined by TP-PCR vs standardized RNAase protection assay in 18 human breast tumours

ER cDNA (Figure 1B) using primers in exons 4 and 6, and exon 7-deleted ER cDNA (Figure 1C), using primers in exons 5 and 8, were measured. The median value for the relative expression of the exon 5-deleted ER for the primary tumours was 23.1% (range 17.3–94.3%) and the median value for the matched lymph node metastases was 31.3% (range 14.9–200%). The scatter plot for these results is shown in Figure 2A. The median relative expression of the exon 7-deleted ER for primary tumours was 65% (range 39.3–184.9%) and the median value for the matched lymph node metastases was 52.5% (range 35.5–126%). The scatterplot of these results is shown in Figure 2B. There were no statistically significant differences in the relative expression of either exon-deleted ER mRNA between primary and concurrent metastatic tumours.

### Comparison of RNAase protection assay and triple-primer PCR assay for determination of the relative expression of clone 4 truncated ER variant mRNA expression

Another frequently expressed ER variant, which would not be detected in the above assays, is the C4 ER mRNA. This variant was previously found to be significantly elevated in a group of primary breast tumours with poor prognostic features that included concurrent lymph node metastases, compared with a group of primary tumours with good prognostic variables that included lack of concurrent nodal metastases (Murphy et al, 1995). Therefore, it was relevant to determine the level of C4 ER variant expression in primary breast tumours and their matched, concurrent lymph node metastases.

In this previous study, we used RNAase protection assays to measure WT and variant ER mRNA expression (Murphy et al, 1995). However, in order to conduct this study using smaller tissue samples (in particular from nodal metastases) and to ensure a close correlation with the histological composition of the tissue, we used a previously described TP-PCR assay (Leygue et al, 1996*b*) to measure the relative expression of C4 ER mRNA. To facilitate comparison of the current data with our earlier study (Murphy et al, 1995), it was necessary to compare the RNAase protection assay with the TP-PCR assay, before proceeding to analyse the primary and secondary breast tumour samples for C4 mRNA expression by TP-PCR.

RNA from 25 human breast tumours, selected to represent a wide range of ER status by ligand-binding assay (Table 1), was

**Table 1** C4 and WT ER mRNA expression in 25 human breast tumours, as determined by RNAase protection assay and TP-PCR

| Sample no. | Ligand binding | RNAase protection | | | TPPCR |
|---|---|---|---|---|---|
| | ER (fmol mg$^{-1}$) | C4 (pg 10 μg$^{-1}$) | WT ER (pg 10 μg$^{-1}$) | C4 (%) | C4 (%) |
| 5 | 0.0 | ND | ND | – | 1.7 |
| 3 | 0.4 | ND | ND | – | 2.6 |
| 1 | 0.9 | ND | ND | – | 3.1 |
| 24 | 1.2 | 6.2 | 105.1 | 5.9 | 3.3 |
| 4 | 1.8 | ND | ND | – | 3.7 |
| 23 | 4.5 | 10.0 | 54.3 | 18.4 | 22.7 |
| 8 | 5.8 | ND | 26.8 | – | 2.8 |
| 7 | 6.3 | ND | 224.6 | – | 3.4 |
| 2 | 8.7 | ND | 9.0 | – | 2.2 |
| 19 | 10.0 | 22.6 | 902.9 | 2.5 | 3.6 |
| 10 | 17.8 | 5.3 | 146.4 | 3.6 | 4.1 |
| 13 | 25.0 | 2.3 | 112.0 | 2.0 | 1.0 |
| 15 | 44.0 | 5.0 | 148.5 | 3.4 | 5.9 |
| 22 | 57.0 | 11.8 | 153.6 | 7.7 | 14.1 |
| 11 | 90.0 | 2.5 | 129.1 | 1.9 | 1.7 |
| 21 | 96.0 | 9.6 | 263.4 | 3.6 | 2.2 |
| 14 | 105.0 | 4.6 | 94.4 | 4.9 | 5.0 |
| 17 | 111.0 | 26.7 | 320.3 | 8.3 | 9.1 |
| 9 | 121.0 | 4.6 | 277.7 | 1.7 | 2.4 |
| 6 | 146.0 | 2.0 | 105.0 | 1.9 | 1.9 |
| 18 | 198.0 | 15.8 | 422.0 | 3.7 | 7.0 |
| 20 | 236.0 | 8.8 | 288.4 | 3.0 | 3.5 |
| 12 | 289.0 | 3.6 | 80.5 | 4.5 | 8.0 |
| 16 | 304.0 | 38.8 | 1440.8 | 2.7 | 3.7 |
| 25 | 311.0 | 83.9 | 3651.0 | 2.3 | 3.2 |

ND, not detected.

analysed in a standardized RNAase protection assay in order to determine the absolute amount of C4 and WT ER mRNAs within each sample. The signals corresponding to C4 and WT ER mRNAs were quantified as described in Materials and Methods. In each assay, known amounts of synthetic WT ER and C4 mRNAs were analysed in parallel in order to establish a standard curve allowing the determination of absolute levels of C4 and WT ER mRNAs, expressed as pg 10 μg$^{-1}$ RNA (Table 1). Because of the very low C4 protected fragment signal (≤ 15 d.p.m.) in seven tumours, it was not possible to determine confidently the absolute amount of C4 mRNA in these samples (not detected, ND). All C4-negative tumours by RNAase protection assay were from tumours with ER values lower than 10 fmol mg$^{-1}$ protein, as determined by ligand-binding assay. The absolute amounts of C4 and WT ER mRNAs in the remaining 18 tumours, as determined by RNAase protection assay, varied from 2 to 83.9 pg 10 μg$^{-1}$ RNA and from 9 to 3651 pg 10 μg$^{-1}$ RNA respectively. For each sample, the C4 mRNA signal was expressed as a percentage of WT ER mRNA signal (Table 1).

C4 ER mRNA relative expression was determined by TP-PCR within the same 25 RNA samples as described in Materials and Methods. Both C4 and WT ER cDNAs signals were detected in all 25 tumours studied, independent of their ER status as determined by ligand-binding assay. C4 and WT ER signals were quantified as described in Materials and Methods. The signal corresponding to C4 was expressed as a percentage of the WT ER signal. Table 1 presents the average of a least two independent TP-PCR experiments. Linear regression analysis (Figure 3) shows a highly significant correlation between C4 mRNA relative expression as

© Cancer Research Campaign 1999

determined by RNAase protection assay (in the 18 tumours in which a C4 signal was detectable) and C4 mRNA relative expression determined by TP-PCR ($r = 0.932$, $P < 0.0001$). Interestingly, an additional band was also observed in most of the samples using the TP-PCR assay (see asterisk, Figure 1D). This band was identified after subcloning and sequencing to be a product of an exon 2-duplicated ER variant mRNA. The intensity of the signal obtained from this exon 2-duplicated ER band paralleled that of the WT ER band, and the co-amplification of the exon 2-duplicated ER variant mRNA using TP-PCR did not interfere with the relationship between TP-PCR and RNAase protection assay.

### Determination of the relative expression of clone 4 truncated ER variant mRNA expression

The above TP-PCR assay was used to compare the relative expression of C4 and WT ER expression in the matched breast cancer samples (Figure 1D). The median relative expression of the C4 ER for the primary tumours was 3.5% (range 1.6–10.5%) and the median value for the matched lymph node metastases was 3.1% (range 1.0–19.4%). A scatterplot of the results is shown in Figure 2C. There is no statistically significant difference in the relative expression of C4 ER variant expression between primary breast tumours and their concurrent lymph node metastases by Wilcoxon rank-sum analysis. Interestingly, although not statistically significant, we found that the median level of C4 expression in ER+ PR– primary tumours, 3.7% (range 2.5–7.9%, $n = 5$), was approximately 50% higher than the median level of C4 expression in ER+ PR+ primary tumours, which was 2.4% (range 1.6–10.5%, $n = 8$). Such a trend would be consistent with our previous results in which C4 expression was higher in PR– primary breast tumours than in PR+ primary tumours.

### DISCUSSION

The data presented in this study provide evidence that both the overall pattern of ER variant expression and the relative level of expression of three individual ER variants are conserved in primary breast tumours and their matched, concurrent lymph node metastases.

The observations presented in this manuscript, showing a conserved pattern and similar relative expression of ER variants between primary tumours and their concurrent lymph node metastases, would be consistent with previous observations that little change of ER status can be found between primary human breast tumours and their concurrent lymph node metastases or their distant metastases (Hahnel and Twaddle, 1985; Robertson, 1996). These findings are not inconsistent with our previously published data, which showed that the relative expression of one ER variant was significantly increased in primary tumours with poor prognostic characteristics, which included having concurrent lymph node metastases, as compared with primary tumours without concurrent lymph node metastases (Murphy et al, 1995). It should be stressed that all the primary tumours in the current study had concurrent lymph node metastases, a major feature of poor prognosis in breast cancer, and most likely resembled our previously described poor prognostic group (Murphy et al, 1995). Therefore in primary tumours that have concurrent lymph node metastases and have detectable levels of C4 ER variant as well as other variant ER mRNAs, mRNA levels do not significantly change between primary tumours and their concurrent lymph node metastases. These data do not, however, shed any light on whether tumours with good prognostic features, as previously described (Murphy et al, 1995), that have a relatively low level of C4 ER variant mRNA subsequently develop higher levels when recurrent disease develops. Although this issue remains to be investigated, our earlier observation of higher relative C4 ER mRNA expression in PR– primary tumours compared with PR+ primary tumours appeared to be conserved in the present cohort, although the numbers were low and the difference did not reach statistical significance. As quantitative differences in the expression of several ER variants have been shown to occur in primary breast tumours compared with normal human breast tissues (Leygue et al, 1996*a, b*), as well as between good vs poor prognosis primary breast tumours, the current data suggest that alterations in ER variant expression and any role this may have in altered oestrogen signal transduction probably occurs early in tumorigenesis and well before the acquisition of the ability to metastasize. This is consistent with previous data supporting the concept of an early involvement of perturbations of oestrogen signal transduction and the development of hormone independence in breast tumorigenesis (Khan et al, 1994; Schmitt, 1995). It remains therefore to be determined if altered ER variant expression can predict tumour recurrence and progression in node-negative breast cancers.

To our knowledge, this study is the first to compare an already established quantitative approach, such as the RNAase protection assay, with an RT-PCR based approach in the study of ER variant mRNA expression. Earlier studies have utilized either the RNAase protection assay or RT-PCR only. Considering the potential clinical relevance of the measurement of the relative level of ER variants with respect to WT ER within human breast tissue samples and the sensitivity of an RT-PCR based approach, such a comparative study was deemed necessary. Furthermore, our data provide validation for comparing previous data obtained using a non-amplification-dependent RNAase protection assay with the current data obtained using an amplification-dependent TP-PCR assay.

The lack of sensitivity of the RNAase protection assay for a subset of tumours with very low (<10 fmol $mg^{-1}$) ER values by ligand-binding assay is an important limiting factor. It effectively means that, in a screening study, ER-negative tumours (< 3 fmol $mg^{-1}$ protein), as well as ER-positive tumours with ER values lower than 10 fmol $mg^{-1}$, as measured by ligand-binding assay, cannot be reliably assessed for C4 ER variant mRNA expression by RNAase protection assay. This, together with the relatively large amount of RNA needed to perform an RNAase protection analysis, severely limits the usefulness of a standardized RNAase protection assay in such screening studies. The low amount of starting material needed, together with the higher sensitivity observed (samples C4 ER variant negative by RNAase protection assay had detectable levels of C4 ER variant and WT ER mRNA by TP-PCR) make TP-PCR an attractive alternative to the RNAase protection assay in studies in which such factors are limiting.

In conclusion, the current investigation extends our previous studies on the relationship of ER variant expression and progression in human breast cancer. The data presented show that both the pattern and level of expression of ER variants are conserved between matched primary breast tumours and their concurrent lymph node metastases. Therefore, any alteration in ER variant expression that could be a marker of altered ER signal transduction and breast cancer progression probably occurs before breast cancer cells acquire the ability to metastasize.

© Cancer Research Campaign 1999

### ACKNOWLEDGEMENTS

This work was supported by the US Army Medical Research and Materiel Command (USAMRMC), grant number DAMD17-95-1-5015. The Manitoba Breast Tumour Bank is supported by funding from the National Cancer Institute of Canada (NCIC). LCM is a Medical Research Council of Canada (MRC) Scientist, PHW is a MRC Clinician-Scientist, EL is a recipient of a USAMRMC Postdoctoral Fellowship (DAMD17-96-1-6174), RH was supported by a University of Manitoba Visiting Scientist Award.

### REFERENCES

Daffada AA, Johnston SR, Nicholls J and Dowsett M (1994) Detection of wild type and exon 5-deleted splice variant oestrogen receptor (ER) mRNA in ER-positive and -negative breast cancer cell lines by reverse transcription/polymerase chain reaction. *J Mol Endocrinol* **13**: 265–273

Daffada AA, Johnston SR, Smith IE, Detre S, King N and Dowsett M (1995) Exon 5 deletion variant estrogen receptor messenger RNA expression in relation to tamoxifen resistance and progesterone receptor/pS2 status in human breast cancer. *Cancer Res* **55**: 288–293

Desai A, Luqmani Y, Coope R, Dagg B, Gomm J, Pace P, Rees C, Thirunavukkarasu V, Shousha S, Groome N, Coombes R and Ali S (1997) Presence of exon 5 deleted oestrogen receptor in human breast cancer: functional analysis and clinical significance. *Br J Cancer* **75**: 1173–1184

Dotzlaw H, Miller T, Karvelas J and Murphy LC (1990) Epidermal growth factor gene expression in human breast cancer biopsy samples: relationship to estrogen and progesterone receptor gene expression. *Cancer Res* **50**: 4204–4208

Dotzlaw H, Alkhalaf M and Murphy LC (1992) Characterization of estrogen receptor variant mRNAs from human breast cancers. *Mol Endocrinol* **6**: 773–785

Dowsett M, Daffada A, Chan C and Johnston S (1997) Oestrogen receptor mutants and variants in breast cancer. *Eur J Cancer* **33**: 1177–1183

Fasco M (1997) Quantitation of estrogen receptor mRNA and its alternatively spliced mRNAs in breast tumor cells and tissues. *Anal Biochem* **245**: 167–178

Green S, Walter P, Kumar V, Krust A, Bornert J, Argos P and Chambon P (1986) Human estrogen receptor cDNA: sequence, expression, and homology to v-erb-A. *Nature* **320**: 134–139

Hahnel R and Twaddle E (1985) The relationship between estrogen receptors in primary and secondary breast carcinomas and in sequential primary breast carcinomas. *Breast Cancer Res Treat* **5**: 155–163

Huang A, Leygue E, Snell L, Murphy LC and Watson P (1997) Expression of estrogen receptor variants mRNAs and determination of estrogen status in human breast cancer. *Am J Pathol* **150**: 1827–1833

Khan SA, Rogers MA, Obando JA and Tamsen A (1994) Estrogen receptor expression of benign breast epithelium and its association with breast cancer. *Cancer Res* **54**: 993–997

Leygue ER, Watson PH and Murphy LC (1996*a*) Estrogen receptor variants in normal human mammary tissue. *J Natl Cancer Inst* **88**: 284–290

Leygue E, Murphy LC, Kuttenn F and Watson P (1996*b*) Triple primer polymerase chain reaction. A new way to quantify truncated mRNA expression. *Am J Pathol* **148**: 1097–1103

Leygue E, Huang A, Murphy LC and Watson P (1996*c*) Prevalence of estrogen receptor variant messenger RNAs in human breast cancer. *Cancer Res* **56**: 4324–4327

Murphy LC and Dotzlaw H (1989) Variant estrogen receptor mRNA species detected in human breast cancer biopsy samples. *Mol Endocrinol* **3**: 687–693

Murphy LC, Hilsenbeck SG, Dotzlaw H and Fuqua SAW (1995) Relationship of clone 4 estrogen receptor variant messenger RNA expression to some known prognostic variables in human breast cancer. *Clin Cancer Res* **1**: 155–159

Murphy LC, Dotzlaw H, Leygue E, Douglas D, Coutts A and Watson P (1997*a*). Estrogen receptor variants and mutations: a review. *J Steroid Biochem Mol Biol* **62**: 363–372

Murphy LC, Leygue E, Dotzlaw H, Douglas D, Coutts A and Watson P (1997*b*) Oestrogen receptor variants and mutations in human breast cancer. *Ann Med* **29**: 221–234

Park W, Choi J, Hwang E and Lee J (1996) Identification of a variant estrogen receptor lacking exon 4 and its coexpression with wild type estrogen receptor in ovarian carcinomas. *Clin Cancer Res* **2**: 2029–2035

Robertson J (1996) Oestrogen receptor: a stable phenotype in breast cancer. *Br J Cancer* **73**: 5–12

Schmitt F (1995) Multistep progression from an oestrogen dependent growth towards an autonomous growth in breast carcinogenesis. *Eur J Cancer* **12**: 2049–2052

*© Cancer Research Campaign 1999* 

## References

[PDF_00507] Daffada A. A., Johnston S. R., Nicholls J., Dowsett M. (1994). Detection of wild type and exon 5-deleted splice variant oestrogen receptor (ER) mRNA in ER-positive and -negative breast cancer cell lines by reverse transcription/polymerase chain reaction.. J Mol Endocrinol.

[PDF_00511] Daffada A. A., Johnston S. R., Smith I. E., Detre S., King N., Dowsett M. (1995). Exon 5 deletion variant estrogen receptor messenger RNA expression in relation to tamoxifen resistance and progesterone receptor/pS2 status in human breast cancer.. Cancer Res.

[PDF_00515] Desai A. J., Luqmani Y. A., Walters J. E., Coope R. C., Dagg B., Gomm J. J., Pace P. E., Rees C. N., Thirunavukkarasu V., Shousha S. (1997). Presence of exon 5-deleted oestrogen receptor in human breast cancer: functional analysis and clinical significance.. Br J Cancer.

[PDF_00523] Dotzlaw H., Alkhalaf M., Murphy L. C. (1992). Characterization of estrogen receptor variant mRNAs from human breast cancers.. Mol Endocrinol.

[PDF_00519] Dotzlaw H., Miller T., Karvelas J., Murphy L. C. (1990). Epidermal growth factor gene expression in human breast cancer biopsy samples: relationship to estrogen and progesterone receptor gene expression.. Cancer Res.

[PDF_00526] Dowsett M., Daffada A., Chan C. M., Johnston S. R. (1997). Oestrogen receptor mutants and variants in breast cancer.. Eur J Cancer.

[PDF_00528] Fasco M. J. (1997). Quantitation of estrogen receptor mRNA and its alternatively spliced mRNAs in breast tumor cells and tissues.. Anal Biochem.

[PDF_00530] Green S., Walter P., Kumar V., Krust A., Bornert J. M., Argos P., Chambon P. (1986). Human oestrogen receptor cDNA: sequence, expression and homology to v-erb-A.. Nature.

[PDF_00536] Huang A., Leygue E. R., Snell L., Murphy L. C., Watson P. H. (1997). Expression of estrogen receptor variant messenger RNAs and determination of estrogen receptor status in human breast cancer.. Am J Pathol.

[PDF_00533] Hähnel R., Twaddle E. (1985). The relationship between estrogen receptors in primary and secondary breast carcinomas and in sequential primary breast carcinomas.. Breast Cancer Res Treat.

[PDF_00539] Khan S. A., Rogers M. A., Obando J. A., Tamsen A. (1994). Estrogen receptor expression of benign breast epithelium and its association with breast cancer.. Cancer Res.

[PDF_00542] Leygue E. R., Watson P. H., Murphy L. C. (1996). Estrogen receptor variants in normal human mammary tissue.. J Natl Cancer Inst.

[PDF_00547] Leygue E., Huang A., Murphy L. C., Watson P. H. (1996). Prevalence of estrogen receptor variant messenger RNAs in human breast cancer.. Cancer Res.

[PDF_00544] Leygue E., Murphy L., Kuttenn F., Watson P. (1996). Triple primer polymerase chain reaction. A new way to quantify truncated mRNA expression.. Am J Pathol.

[PDF_00555] Murphy L. C., Dotzlaw H., Leygue E., Douglas D., Coutts A., Watson P. H. (1997). Estrogen receptor variants and mutations.. J Steroid Biochem Mol Biol.

[PDF_00550] Murphy L. C., Dotzlaw H. (1989). Variant estrogen receptor mRNA species detected in human breast cancer biopsy samples.. Mol Endocrinol.

[PDF_00552] Murphy L. C., Hilsenbeck S. G., Dotzlaw H., Fuqua S. A. (1995). Relationship of clone 4 estrogen receptor variant messenger RNA expression to some known prognostic variables in human breast cancer.. Clin Cancer Res.

[PDF_00558] Murphy L. C., Leygue E., Dotzlaw H., Douglas D., Coutts A., Watson P. H. (1997). Oestrogen receptor variants and mutations in human breast cancer.. Ann Med.

[PDF_00561] Park W., Choi J. J., Hwang E. S., Lee J. H. (1996). Identification of a variant estrogen receptor lacking exon 4 and its coexpression with wild-type estrogen receptor in ovarian carcinomas.. Clin Cancer Res.

[PDF_00564] Robertson J. F. (1996). Oestrogen receptor: a stable phenotype in breast cancer.. Br J Cancer.

[PDF_00566] Schmitt F. C. (1995). Multistep progression from an oestrogen-dependent growth towards an autonomous growth in breast carcinogenesis.. Eur J Cancer.

